# Gender differences in patients presenting with non-ST segment elevation myocardial infarction in the STAR registry

**DOI:** 10.1186/s43044-021-00181-6

**Published:** 2021-06-22

**Authors:** Abdulhalim Jamal Kinsara, Yasser M. Ismail

**Affiliations:** Department of Cardiology, King Saud Bin Abdulaziz University for Health Sciences, College of medicine, King Abdullah International Medical Research Center, Ministry of National Guard-Health Affairs, Jeddah, Saudi Arabia

**Keywords:** Gender, NSTEMI, STAR registry

## Abstract

**Background:**

In most acute coronary artery (ACS) related literature, the female gender constitutes a smaller proportion. This study is based on gender-specific data in the Saudi Acute Myocardial Infarction Registry Program (STARS-1 Program). A prospective multicenter study, conducted with patients diagnosed with ACS in 50 participating hospitals.

**Results:**

In total, 762 (34.12%) patients were diagnosed with non-ST segment elevation myocardial infarction. Of this group, only 164 (21.52%) were women. The mean age (64.52 ± 12.56 years) was older and the mean body mass index (BMI) was higher (30.58 ± 6.23). A significantly proportion was diabetic or hypertensive; however, a smaller proportion was smoking. Hyperlipidemia was present in 48%. The history of angina/MI/stroke and revascularization was similar, except for renal impairment. The presentation was atypical as only 70% presented with chest pain, and the rest with shortness of breath or epigastric pain. At presentation, the female group were more tachycardiac, had higher blood pressure, and a higher incidence of being in class 11-111 Killip heart failure. Only 32% had a normal systolic function, and the majority had either mild or moderate systolic dysfunction.

In particular, the rate of percutaneous coronary intervention was similar. The in-hospital mortality was similar (5%), with more women diagnosed with atrial fibrillation and heart failure at follow-up.

**Conclusions:**

Women had a higher prevalence of risk factors affecting the presentation and morbidity but not mortality. Improving these risk factors and the lifestyle is a priority to improve the outcome and decrease morbidity.

## Background

Gender differences in scientific publications have always been a concern. Such differences may adversely affect the clinical features, management, and most importantly, the outcome [1]. Exploring gender-related data might positively affect the prognosis. A continuous focus on gender differences is important as it improves our understanding [1]. Saudi Arabia, as a developing country, faced the same challenge and this work presents a detailed analysis of data from 50 centers across the country [2]. The data was extracted from secondary and tertiary hospitals and from different healthcare sectors, including hospitals with and without a catheter laboratory.

## Methods

The STAR is a prospective study of all patients presenting with acute coronary syndrome (ACS) to an emergency department at 50 hospitals across Saudi Arabia. The details of the study have been described previously [2].

The design was a prospective, multi-center, recruited all consecutive AMI (STEMI or NSTEMI) admissions. All relevant data were gathered at admission, 1-month and 1-year follow-up.

The recruiting hospitals were both the one who had catheterization laboratory or not and included various health sectors in Saudi Arabia.

This study aimed to see the difference in the management strategy and the outcome of treatment among male and female patients. This snap shot of two groups will assess if temporal changes in AMI care between genders that were noted among different societies in different studies.

## Results

In total, 762 (34.12%) patients were diagnosed with non-ST segment elevation myocardial infarction. Of this group, only 164 (22.52%) were women. The mean age of the group (64.52 ± 12.56 years) was older than the male group and the mean body mass index (BMI) was higher (30.58 ± 6.23). A significantly higher proportion of the female group was diabetic or hypertensive; however, a smaller proportion was smoking. Hyperlipidemia was not significant between the two groups, although present in almost half (48%) of the female group. The history of angina/MI/stroke and revascularization was similar, except for renal impairment. The presentation was atypical compared to the male group as only 70% presented with chest pain, and the rest with shortness of breath or epigastric pain. At presentation, the female group were more tachycardiac, had higher blood pressure, and a higher incidence of being in class 11-111 Killip heart failure. Only 32% had a normal systolic function, and the majority had either mild or moderate systolic dysfunction (Table 1).

**Table 1:** Epidemiological data and presentation characteristics of NSTEMI patients by gender

	Male *N* = 598 (78.48%)	Female *N* = 164 (21.52%)	Total *N* = 762	*P* value
Variable#				
Age	56.68 ± 13.30	64.52 ± 12.56	58.37 ± 13.53	<. 001
Saudi	322 (53.85%)	137 (83.54%)	459 (60.24%)	<. 001
Ethnicity				<. 001
Arab	427 (71.40%)	155 (94.51%)	582 (76.38%)	
South Asian	152 (25.42%)	8 (4.88%)	160 (21.00%)	
Others	19 (3.18%)	1 (0.61%)	20 (2.62%)	
BMI	28.58 ± 5.33	30.58 ± 6.23	29.01 ± 5.59	<. 001
History of angina	202 (33.78%)	68 (41.46%)	270 (35.43%)	0.068
History of MI	139 (23.24%)	36 (21.95%)	175 (22.97%)	0.727
History MI angina	241 (40.30%)	74 (45.12%)	315 (41.34%)	0.267
History of PCI	109 (18.23%)	30 (18.29%)	139 (18.24%)	0.985
History of CABG	32 (5.35%)	8 (4.88%)	40 (5.25%)	0.810
History of heart failure	54 (9.03%)	25 (15.24%)	79 (10.37%)	0.021
History of stroke	30 (5.02%)	11 (6.71%)	41 (5.38%)	0.395
History of chronic renal failure	49 (8.19%)	24 (14.63%)	73 (9.58%)	0.013
DM	334 (55.85%)	132 (80.49%)	466 (61.15%)	<. 001
HTN	355 (59.36%)	140 (85.37%)	495 (64.96%)	<. 001
Hypercholesterolemia	245 (40.97%)	79 (48.17%)	324 (42.52%)	0.098
Current/ex-smoking	343 (57.36%)	8 (4.88%)	351 (46.06%)	<. 001
Chief complaint				0.002
Chest pain	507 (84.78%)	117 (71.34%)	624 (81.89%)	
SOB/fatigue	58 (9.70%)	33 (20.12%)	91 (11.94%)	
Epigastric/shoulder/back/neck pain	21 (3.51%)	10 (6.10%)	31 (4.07%)	
Cardiac arrest	2 (0.33%)	1 (0.61%)	3 (0.39%)	
Others	10 (1.67%)	3 (1.83%)	13 (1.71%)	
First medical contact	163 (27.26%)	27 (16.46%)	190 (24.93%)	0.005
Visited emergency department	142 (87.12%)	25 (92.59%)	167 (87.89%)	0.419
Clinic doctor	27 (16.56%)	4 (14.81%)	31 (16.32%)	0.820
Visited a pharmacy	3 (1.84%)	1 (3.70%)	4 (2.11%)	0.532
Transferred by Saudi Red Crescent	15 (2.51%)	7 (4.27%)	22 (2.89%)	0.233
HR (bpm) upon arrival	84.01 ± 19.36	91.64 ± 18.94	85.65 ± 19.51	<. 001
SBP (mm Hg) upon arrival	135.8 ± 25.56	144.3 ± 30.82	137.6 ± 26.99	<.001
HR > 100 bpm	93 (15.55%)	42 (25.61%)	135 (17.72%)	0.003
SBP < 90 mm Hg	8 (1.34%)	3 (1.83%)	11 (1.44%)	0.640
Cardiac arrest upon arrival				
Cardiac arrest upon arrival	7 (1.17%)	1 (0.61%)	8 (1.05%)	0.532
CHF Killip class				
Class I	507 (84.78%)	109 (66.46%)	616 (80.84%)	< .001
Class II/III	84 (14.05%)	53 (32.32%)	137 (17.98%)	
IV	7 (1.17%)	2 (1.22%)	9 (1.18%)	
Echo options				
Normal LV systolic function (EF > 50%)	237 (43.33%)	53 (35.33%)	290 (41.61%)	0.025
Mild LV systolic dysfunction (EF 40-50%)	164 (29.98%)	52 (34.67%)	216 (30.99%)	
Moderate LV systolic dysfunction (EF 30-40%)	88 (16.09%)	36 (24.00%)	124 (17.79%)	
Severe LV systolic dysfunction (EF < 30%)	58 (10.60%)	9 (6.00%)	67 (9.61%)	
ECG done/transferred				
ECG done/transferred	340 (56.86%)	68 (41.46%)	408 (53.54%)	< .001
Arterial access				
Femoral	124 (46.62%)	28 (59.57%)	152 (48.56%)	0.101
Radial	142 (53.38%)	19 (40.43%)	161 (51.44%)	

Guideline-directed medical therapy was not different between the two groups, except for the initiation of a beta-blocker on admission. In particular, the rate of percutaneous coronary intervention (PCI) was similar (Tables 2 and 3).

**Table 2:** Medication at admission

Medications 24 h	Male	Female	Total	*P* value
Aspirin	592 (99.00%)	163 (99.39%)	755 (99.08%)	0.640
Clopidogrel	533 (89.13%)	154 (93.90%)	687 (90.16%)	0.069
Prasugrel	3 (0.50%)	0 (0.00%)	3 (0.39%)	0.363
Ticagrelor	59 (9.87%)	5 (3.05%)	64 (8.40%)	0.005
Beta blockers	531 (88.80%)	130 (79.27%)	661 (86.75%)	0.001
ACEI/ARB	462 (77.26%)	132 (80.49%)	594 (77.95%)	0.377
Statins	570 (95.32%)	159 (96.95%)	729 (95.67%)	0.363
Aldosterone inhibitor (spironolactone)	54 (9.03%)	18 (10.98%)	72 (9.45%)	0.451
Heparins UH/LMWH	552 (92.31%)	148 (90.24%)	700 (91.86%)	0.392
GP_2b3a_inhibitors	97 (16.22%)	15 (9.15%)	112 (14.70%)	0.023
Insulin	284 (47.49%)	114 (69.51%)	398 (52.23%)	< .001
Oral hp agents	74 (12.37%)	32 (19.51%)	106 (13.91%)	0.019

**Table 3:** Medication at discharge

Medications discharge	Male	Female	Total	*P* value
Aspirin	566 (98.26%)	153 (98.08%)	719 (98.22%)	0.875
Clopidogrel	483 (83.85%)	132 (84.62%)	615 (84.02%)	0.818
Ticagrelor	61 (10.59%)	12 (7.69%)	73 (9.97%)	0.284
Beta blockers	529 (91.84%)	136 (87.18%)	665 (90.85%)	0.073
ACEI/ARB	457 (79.34%)	126 (80.77%)	583 (79.64%)	0.694
Statins	562 (97.57%)	151 (96.79%)	713 (97.40%)	0.589
Aldosterone inhibitor (spironolactone)	65 (11.28%)	25 (16.03%)	90 (12.30%)	0.110
Oral anticoagulant warfarin/dabigatran	18 (3.13%)	9 (5.77%)	27 (3.69%)	0.120
Insulin	179 (31.08%)	89 (57.05%)	268 (36.61%)	< .001
Oral hypoglycemic	166 (28.82%)	54 (34.62%)	220 (30.05%)	0.161

Overall, the in-hospital mortality was similar (5%), and more women were diagnosed with atrial fibrillation and heart failure at follow-up (Table 4). No difference were noted between the groups in recurrent ischemia, recurrent MI, cardiogenic shock, VTVF arrest, stroke, or major bleeding.

**Table 4:** Outcome differences of NSTEMI patients by gender

In-hospital outcomes	Male	Female	Total	*P* value
Recurrent ischemia	70 (11.71%)	28 (17.07%)	98 (12.86%)	0.069
Recurrent MI	34 (5.69%)	14 (8.54%)	48 (6.30%)	0.183
Atrial fibrillation/flutter	28 (4.68%)	16 (9.76%)	44 (5.77%)	0.014
Heart failure	65 (10.87%)	34 (20.73%)	99 (12.99%)	< .001
Cardiogenic shock	32 (5.35%)	10 (6.10%)	42 (5.51%)	0.711
VTVF arrest	31 (5.18%)	13 (7.93%)	44 (5.77%)	0.182
Stroke	11 (1.84%)	3 (1.83%)	14 (1.84%)	0.993
Major bleeding	5 (0.84%)	1 (0.61%)	6 (0.79%)	0.771
In-hospital mortality	22 (3.68%)	8 (4.88%)	30 (3.94%)	0.484

## Discussion

Women are still underrepresented in ACS-related literature. Based on the gender distribution in the country, we expected a larger proportion, not only 22% as reported in the current study. Because the data were extracted from the registry, collection bias played no role in the study. Possible explanations could be that the women had ACS but did not reach medical attention or they were incorrectly diagnosed due to their atypical symptoms. Both are major concerns that require further investigation and governmental attention, as previously reported [3, 4].

We also noted the high incidence of diabetes and hypertension in the female group. Both are modifiable risk factors that reflect the need to modify the lifestyle. The medical staff, especially at emergency rooms and paramedics, should be aware of the atypical presentation. The Red Crescent was under-utilized and a mass educational intervention is urgent.

Although the findings are rising alarms, the effort did not match the challenge [5]. The issue is global, extending from east to west [6–8].

## Conclusion

Women are underrepresented, frequently had an atypical presentation and presented late. Risk factors are highly prevalent and need immediate attention. It should be stated that when they did present for medical attention, they received appropriate guideline-directed medical therapy and PCI.

## Data Availability

Via IRB office

## Abbreviations

ACS: Acute coronary artery
STARS-1 Program: Saudi Acute Myocardial Infarction Registry Program
NSTEMI: Non-ST segment elevation myocardial infarction
BMI: Body mass index
PCI: Percutaneous coronary intervention
